# Jarosite formation in deep Antarctic ice provides a window into acidic, water-limited weathering on Mars

**DOI:** 10.1038/s41467-020-20705-z

**Published:** 2021-01-19

**Authors:** Giovanni Baccolo, Barbara Delmonte, P. B. Niles, Giannantonio Cibin, Elena Di Stefano, Dariush Hampai, Lindsay Keller, Valter Maggi, Augusto Marcelli, Joseph Michalski, Christopher Snead, Massimo Frezzotti

**Affiliations:** 1 Department of Environmental and Earth Sciences, University of Milano-Bicocca, 20126 Milan, Italy; 2 INFN, section of Milano-Bicocca, 20126 Milan, Italy; 3 NASA Johnson Space Center, Houston, TX 77058 USA; 4 Diamond Light Source, Harwell Science and Innovation Campus, Didcot, OX11 0DE UK; 5 Department of Physical, Earth and Environmental Sciences, University of Siena, 53100 Siena, Italy; 6 Laboratori Nazionali di Frascati, Istituto Nazionale di Fisica Nucleare, 00044 Frascati, Italy; 7 Rome International Center for Materials Science - Superstripes, 00185 Rome, Italy; 8 Department of Earth Sciences, University of Hong Kong, Hong Kong, Hong Kong; 9 Jacobs, NASA Johnson Space Center, Houston, TX 77058 USA; 10 Department of Science, University Roma Tre, Rome, Italy

**Keywords:** Cryospheric science, Geochemistry, Inner planets, Mineralogy, Geochemistry

## Abstract

Many interpretations have been proposed to explain the presence of jarosite within Martian surficial sediments, including the possibility that it precipitated within paleo-ice deposits owing to englacial weathering of dust. However, until now a similar geochemical process was not observed on Earth nor in other planetary settings. We report a multi-analytical indication of jarosite formation within deep ice. Below 1000 m depth, jarosite crystals adhering on residual silica-rich particles have been identified in the Talos Dome ice core (East Antarctica) and interpreted as products of weathering involving aeolian dust and acidic atmospheric aerosols. The progressive increase of ice metamorphism and re-crystallization with depth, favours the relocation and concentration of dust and the formation of acidic brines in isolated environments, allowing chemical reactions and mineral neo-formation to occur. This is the first described englacial diagenetic mechanism occurring in deep Antarctic ice and supports the ice-weathering model for jarosite formation on Mars, highlighting the geologic importance of paleo ice-related processes on this planet. Additional implications concern the preservation of dust-related signals in deep ice cores with respect to paleoclimatic reconstructions and the englacial history of meteorites from Antarctic blue ice fields.

## Abstract (web summary)

The authors report in-situ formation of jarosite witin the Talos Dome ice core (East Antarctica) and show that this ferric-potassium sulfate mineral is present in ice deeper than 1000 meters and progressively increases with depth. This has implications for the presence and formation mechanisms of jarosite observed on Mars.

## Introduction

Jarosite, a ferric-potassium hydroxide sulfate [KFe^3+^_3_(SO_4_)_2_(OH)_6_], was firstly hypothesized to be a common mineral on Mars by Burns^1^ despite its rareness on Earth. In 2004 the Opportunity rover reported widespread jarosite at Meridiani Planum^2^, confirming Burns’ prediction, but the geological context where jarosite was found – in fine-grained sediments within layered formations – was difficult to interpret. Since then, the mineral has been repeatedly identified on Mars^3–5^ and has been regarded as evidence for the occurrence of liquid water^6^ because on Earth jarosite forms as the result of low-temperature acidic-oxidative weathering of iron-bearing minerals in water-limited settings^7^.

Limited water is not only necessary for the formation of jarosite, but it is critical to its long-term preservation. Experimental evidence shows that when the local water to rock ratio exceeds 10, jarosite is transformed into goethite^8,9^. The occurrence of jarosite on Mars has thus been interpreted as an indication that weathering fluids have been active over local scales and for a geologically short time interval^10^. An apparent paradox concerns the lithology of the protolith in which jarosite occurs, which is composed of mafic (basaltic) volcanic material^11,12^. The interaction between basalt and acidic solutions has a neutralizing effect incompatible with jarosite formation^13^. The paradox is solved if the interaction occurs in environments isolated from bedrock, where water–rock interaction is limited and low pH is maintained during the diagenesis^14^.

Most of the hypotheses about the formation of Martian jarosite involve the interaction between acidic fluids and weathered sediments in transient lacustrine-evaporative basins similar to Earth playas^3,5,11^ or volcanic settings, such as fumaroles^12^. An alternative proposal predicted that jarosite formation would occur during weathering of mafic dust or fine-grained ash trapped in massive ice, but this process had not been observed until now^14,15^. The ice-weathering model suggested that the interior of ice deposits promotes acidic weathering of dust through cryo-concentration of sulfur-rich volcanic aerosols, leading to jarosite precipitation^13,14^. Experimental evidence has since shown that the weathering rate of basalt-related minerals is elevated at cryogenic temperatures^16^, in accordance with the identification of jarosite as a weathering product in Antarctica^17^. But the englacial formation of jarosite is still a speculation. Until this work, Antarctic jarosite has been found in rock varnishes and weathering rinds formed on the surface of erratics^17^ and of meteorites collected at blue ice fields^18,19^, or in soils developed from sulfide-rich rocks^20^, but not in englacial environments.

Here we report the identification of jarosite in deep Antarctic ice, which we hypothesize as authigenic, resulting from the interaction between aeolian dust and acidic species trapped in the ice. The identification of the mineral is confirmed by several evidences, gathered through the application of different and independent techniques. Our results show that weathering and englacial diagenesis is possible deep inside thick ice, potentially affecting the climatic interpretation of dust records in deep ice cores. Given the similarities between our hypothesis and the Martian ice-weathering model, our findings support that ice-mediated weathering is a viable geological mechanism for jarosite formation on Mars, with major implications for past chemical weathering processes on that planet.

## Results and discussion

### The Talos Dome ice core

**Fig. 1:**
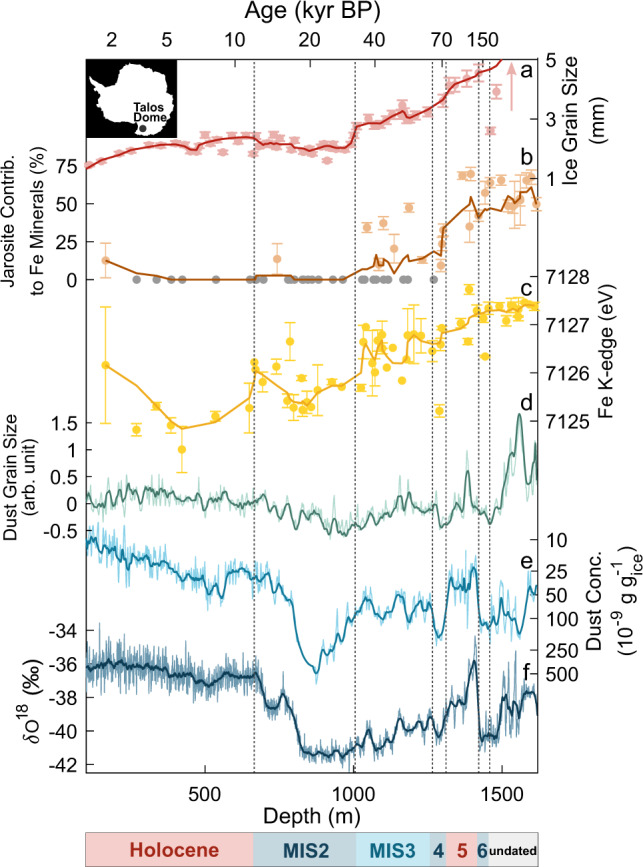
Records from the Talos Dome ice core. **a** Ice-grain size; data from Montagnat et al.^24^. The arrow indicates the very large ice crystals (larger than 40 cm, observed but not quantified) below the depth of 1481 m. **b** Jarosite relative contribution with respect to Fe minerals found in TALDICE dust; grey dots refer to samples where jarosite was not identified, error bars correspond to standard deviations. **c** Energy position of the K-edge X-ray absorption line of the iron fraction within TALDICE dust, error bars correspond to standard deviations. **d** Dust grain size index; developed to highlight the granulometric anomalies observed in dust from the deepest part of TALDICE. **e** Concentration of insoluble mineral dust in ice samples along TALDICE: part of the data from Albani et al.^66^. **f** TALDICE δ^18^O record; data from Stenni et al.^21^. The AICC2012 chronology^22,23^ has been used to prepare the figure. The lower bar refers to the Marine Isotopic Stages (MIS) covered by TALDICE.

The 1620 m long TALDICE (Talos Dome Ice Core 72°49′S, 159°11′E; 2315 m a.s.l.) has been retrieved from a peripheral ice dome of the East Antarctic Plateau^21^ and dated up to 1439 m deep (along the text we refer to “ice depth” to indicate the distance between the ice sheet surface and the considered point into the ice sheet), where the estimated ice age is ~153,000 years before present (BP)^22,23^ (Fig. 1). The deeper part of the core is believed to contain climatic information extending to 250,000 years BP and possibly beyond^21^. Below 1439 m depth, mm-scale visible layers (volcanic ash and cloudy bands) are progressively tilted and folded, and deeper than 1528 m they disappear. In addition, below 1590 m very large ice-crystals (40–50 cm), indicative of ice metamorphism, have been observed^24^ (Fig. 1a). Ice flow disturbances and anomalous ice-crystal growth in the bottom sections of TALDICE have been related to the presence of an irregular bedrock consisting in buried mesas interrupted by canyons, which disturb the ice-flow and the ice stratigraphy^24^. Multiple lines of evidence suggest that throughout the late Quaternary the aeolian dust deposited at Talos Dome was mostly local, in particular during interglacials, when the arrival of dust from remote sources was suppressed^25^. The most important local sources of dust consist in high-elevation ice-free doleritic-basaltic outcrops^25,26^ and in tephra from proximal Antarctic volcanoes^27^. The influence of volcanic activity at Talos Dome is notable. More than 100 volcanic horizons have been identified along the core, with a frequency one order of magnitude greater with respect to what is observed at Dome C or Vostok, located at higher elevation in inner East Antarctica^28^. In addition, the aeolian reworking of volcanic deposits provides a background of volcanic tephra in the atmosphere which is also found in TALDICE^27^.

### Identification of jarosite in Antarctic ice

Most of this work is based on the identification of jarosite in dust from the deep part of TALDICE. Highlighting and quantifying the presence of jarosite within µg-size samples is analytically challenging. Evidences have been gathered through multiple techniques, so as to obtain robust and reliable results. Jarosite minerals have been observed through Scanning Electron Microscopy coupled with Energy Dispersive X-ray spectroscopy (SEM-EDX) as micrometric concretions whose shape and composition resemble jarosite. X-ray absorption spectroscopy has allowed to reconstruct the depth profile of jarosite in TALDICE, exploiting Fe speciation and coordination and a quantitative comparison with mineral standards. Scanning Transmission Electron Microscopy (STEM) and EDX, through diffraction spacings and stoichiometry, have brought a further quantitative confirmation of jarosite presence.

The K-edge absorption energy of the Fe fraction of mineral dust from TALDICE increases with depth (Fig. 1c), pointing to a progressive Fe oxidation^29^. Samples from the 0–1000 m (*n* = 20), 1000–1300 m (*n* = 18) and 1300–1620 m (*n* = 16) depth intervals present average Fe K-edge absorption energies of 7125.5(0.5), 7126.5(0.4) and 7127.2(0.3) eV (standard deviation), respectively. Considering that the positive shift between Fe^2+^ and Fe^3+^ is 4 eV, the one observed along TALDICE corresponds to a 30–40% increase of Fe^3+^^29^, in agreement with the absolute determination of Fe oxidation in TALDICE dust. Between 0 and 1000 m, Fe in mineral dust consists in a 70–30% mix of Fe^3+^–Fe^2+^, while below 1500 m only Fe^3+^ is detected^30^. The trend is indicative of in situ oxidative weathering and involves the core throughout its length, regardless of the climate-related oscillations observed in the stable isotope and dust concentration records (Fig. 1c, e, f). This suggests that Fe oxidation is a signal mostly related to non-climatic processes. Below the depth of 1500 m, the absorption energy presents a plateau at about 7127 eV, consistent with the complete oxidation of Fe. Another evidence of weathering comes from the identification of jarosite within TALDICE dust (Fig. 1b). Above the depth of 1033 m, only 1 of 22 samples presents evidence of jarosite occurrence. Fe K-edge absorption spectra of shallow samples are in fact reproduced using common iron bearing minerals, in particular oxides (mostly goethite, a major component of Earth atmospheric mineral dust^31,32^), but also silicates (Supplementary Fig. 1). At greater depth, a convex feature between 7135 and 7142 eV, indicative of jarosite^30^, appears in X-ray absorption (XAS) spectra (Supplementary Figs. 1 and 2) and accordingly between 1033 and 1500 m the contribution of this mineral increases and becomes dominant, meaning that more than 50 % of the information from Fe-related XAS spectra is reproduced by jarosite. Below 1500 m jarosite contribution maintains a stable value with a mean of 54(8)% (standard deviation), similar to the Fe oxidation pattern observed at these depths, which also reaches a high and relatively stable value. The agreement between the records is expected given that jarosite is a ferric mineral.

**Fig. 2:**
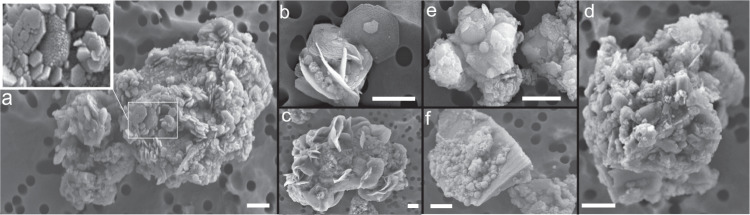
The morphology of mineral grains in deep TALDICE investigated through SEM. Samples were retrieved from TALDICE sections at the depth of 1560 m (**a**, **d**, **e**, **f**) and 1534 m (**b**, **c**). Scale bar: 1 µm.

**Fig. 3:**
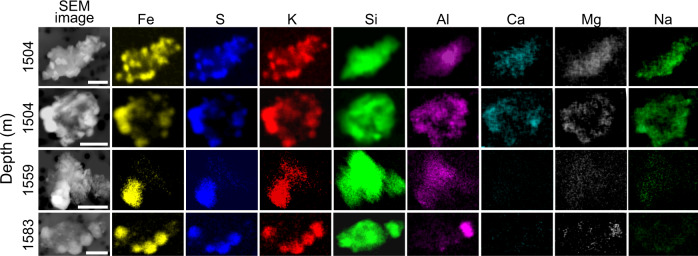
Elemental maps obtained through SEM-EDX of dust grains from deep TALDICE ice. Scale bar: 2 µm.

**Fig. 4:**
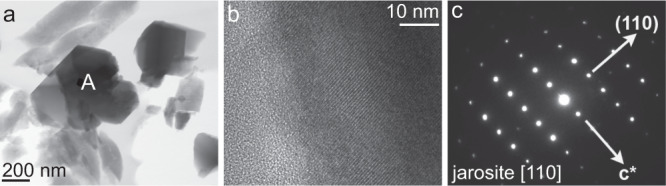
STEM on TALDICE dust. **a** A STEM image of dust particles. **b** A selected area of electron diffraction (SAED) pattern from the crystal labelled A in **a**. **c** The SAED pattern with highlighted [−110] zone axis of jarosite.

The morphology and elemental composition of mineral dust from sections of TALDICE deeper than 1500 m has been investigated through SEM-EDX (Fig. 2). Weathering features are observed, including the presence of micrometric to sub-micrometric-sized precipitates ranging from crystalline minerals (Fig. 2a, b, d) to globular concretions presenting irregular surface cracks, poorly ordered aggregates (Fig. 2c, e, f) and scarcity of sharp edges. Only a few volcanic glasses covered by precipitates are recognized (Fig. 2e). Among the precipitates, hexagonal platelets are frequently observed, either as independent (Fig. 2a) or grouped crystals (Fig. 2b, d), a habit compatible with the trigonal system of jarosite^33^. The morphology of TALDICE deep particles exhibits differences with the ones from the upper core, consisting in micrometric mono-mineral particles and volcanic glasses characterized by flat surfaces and sharp corners, indicative of poor chemical weathering (Supplementary Fig. 3). Elemental maps of individual grains show that precipitates are composed of Fe, S and K, corresponding to jarosite constituents, while grain cores mostly consist in Si or Si and Al, with minor traces of Ca, Mg and Na (Fig. 3). A definitive confirmation that precipitates in deep TALDICE consist of jarosite comes from STEM-EDX applied on two mineral grains from TALDICE samples 1559 m deep. Results confirm the presence of jarosite as bladed crystals with diffraction spacings and chemical composition consistent with jarosite (Fig. 4). The examined crystals are nearly pure KFe^3+^_3_(SO_4_)_2_(OH)_6_, as supported by EDX quantitative results (see the Supplementary Information).

### Englacial chemical weathering of mineral dust in deep ice

Physico-chemical signals are preserved for hundreds of thousands of years within ice^34^. Climatic proxies, such as δ^18^O and dust concentration, are correlated in ice cores and show coherent oscillations during glacial/interglacial cycles^35^. This is confirmed by TALDICE data, where their linear R-squared is 0.75 when considering the depth interval 0–1400 m (Supplementary Fig. 6). In contrast, Fe oxidation and jarosite abundance are not coupled with δ^18^O (R-squared <0.02) and lack of climate-related fluctuations. They are correlated with the increasing size of ice crystals along the core (R-squared = 0.55 and 0.49, respectively), which is primarily driven by ice metamorphism and not by climate^36^. The possibility that jarosite observed in the deep part of TALDICE comes from the bedrock is discarded, since there are no other evidences about the presence of bedrock inclusions. Only an extreme ice-folding could explain the presence of bedrock inclusions hundreds of meters above the ice-bedrock interface, but this is incompatible with the preservation of ice stratigraphy up to 1560 m deep^21^.

Based on our understanding of the environmental conditions of deep ice, we interpret jarosite as the product of englacial weathering. Its formation requires acidic conditions, a limited activity of liquid water and the presence of Fe-bearing materials^7^. The deep part (>1000 m) of TALDICE can meet such requirements. Deep ice is affected by the progressive re-crystallization of ice grains^36–38^. A consequence of re-crystallization is that impurities incompatible with the ice lattice accumulate at ice grain junctions^39,40^ or within intra-grain µ-inclusions^37,41^. Atmospheric sulfates and sulfuric acid are strongly affected by remobilization in ice^39,42^. They are easily concentrated in isolated environments forming solutions whose eutectic temperature is below the pressure melting point of deep ice, allowing for the presence of acidic liquid layers at grain boundaries in the form of localized brines^37–39,42,43^. In deep ice, as a result of the increased temperature and ice metamorphism, the concentration and mobilization of impurities allows for the mixing of soluble and insoluble species, and for their interaction through englacial acid–base reactions^37,41,44,45^. Such small-scale environments promote the local increase of acidity and the chemical weathering of aeolian dust^41^. Dust particles deposited at Talos Dome have a basaltic/doleritic signature and are rich in Fe^26,27^, making deep TALDICE an environment suitable for jarosite precipitation. An additional source of acidity to the deep englacial environment could come from the oxidation of the pyrite present in TALDICE dust. Pyrite is a relatively common secondary mineral in the Victoria Land region^46,47^ and has been recognized in shallow TALDICE dust samples as an accessory mineral (see the Supplementary Information). The degradation of pyrite through the oxidation of Fe^2+^ to Fe^3+^ and the production of sulfuric acid is a common weathering reaction in glacial systems^48^ and could contribute to the local increase of acidity in the englacial environment. The positive shift of the Fe K-edge absorption recognized along the core (Fig. 1c), supports this scenario, since it is indicative of the progressive oxidation of Fe with depth.

The identification of secondary ferric precipitates in deep ice is not completely new, evidences were reported from the two EPICA ice cores: De Angelis et al.^37^ observed micrometric Fe-oxides precipitates within the Dome C ice core while Eichler et al.^41^ interpreted a few micro-inclusions in the Dronning Maud Land ice core as jarosite.

The acidic weathering of basalt in a closed system produces jarosite and amorphous silica^6,13^. This matches the observed composition of the grains observed in deep TALDICE. Jarosite is attached to grains composed almost entirely of Si with detectable Al (Fig. 3), a signature compatible with amorphous silica with substituted Al. The distribution of other elements further supports chemical weathering in deep TALDICE: Mg, Ca and Na are almost absent from jarosite precipitates and scarce within Si-rich grains. This is revealed by SEM results (Fig. 3) and by the previous elemental characterization of TALDICE mineral dust^30^. The scarcity of these elements, common constituents of minerals, suggests that reaction with acidic species leads to the formation of soluble compounds which were removed during the preparation of samples through filtration. This is supported by the identification in deep ice cores of soluble Mg, Ca and Na sulfates^41,43,45^ and by their direct observation in TALDICE when ice is sublimated rather than being filtrated^49^. Weathering of minerals also impacts the grain size of insoluble dust in deep TALDICE. This is shown by the dust grain size index, developed to distinguish upper and deeper TALDICE dust through granulometric features (Fig. 1d). Dust from deep TALDICE presents an excess of large particles and a lack of fine ones with respect to typical dust from Antarctic ice cores (Supplementary Fig. 7), as highlighted by the index which presents positive values only in the bottom part of TALDICE (see the Supplementary Information for details). Such anomalies can relate to the chemical aggregation of mineral particles resulting from jarosite englacial precipitation. This mineral is in fact known for acting as a cement during weathering, favoring the aggregation of particles^33^. We observe that below 1400 m, the linear R-squared between δ^18^O and dust decreases from 0.75 to 0.44 (Supplementary Fig. 6), pointing to a degradation of the climatic significance of the TALDICE dust record.

### Antarctic implications

Jarosite formation in TALDICE constrains the physico-chemical environment of deep Antarctic ice. The bare titration of Antarctic meltwater reveals a mild acidity because of the presence of acid atmospheric species (pH between 5.5 and 6^50^), but jarosite formation and preservation requires a pH lower than 4^6,51^. The only process which can explain how in deep ice such acid conditions are found, at least locally, is the concentration of acids mediated by ice re-crystallization and possibly by the oxidation of the pyrite fraction originally present in the dust deposited at Talos Dome^48^. Another important point concerns the occurrence of liquid water in the form of brines. Only a few direct lines of evidence for its presence in ice cores are available^42^ although questioned^41^, and theoretical supports exist^43^. The discrepancy could arise because while acidic brines are stable at pressure and temperature conditions found in deep ice sheets, measurements on ice cores are carried under different conditions, in particular for pressure. Yet, the occurrence of jarosite, whose formation requires liquid water, is a strong evidence for the actual, but localized, occurrence of acidic brines in deep ice, likely in correspondence of ice grain junctions or at intra-grain micro-inclusions. Our data show that once jarosite has formed, it is relatively stable in the englacial environment – at least considering the depth interval covered by TALDICE – since its contribution increases from 1000 to 1400 m deep (corresponding to a time interval of ~100,000 years) and then remains almost stable (Fig. 1b). This is a further confirmation that the occurrence of liquid water in deep ice sheets is only local and takes place in the form of highly saline and acid fluids. If the presence of water was larger and its solute content lower, jarosite would not be stable and would convert into goethite^8–10,13^. Further studies involving deeper ice cores, where calcium carbonate has been detected and suggests less acidic conditions^37,38^, will be useful to assess if the englacial stability field of jarosite is limited to a particular ice depth interval.

Jarosite is present in TALDICE deeper than 1000 m, suggesting that its formation is associated with a threshold (Fig. 1b). Since jarosite forms also at the Earth surface, high pressure is likely not the limiting factor^7^. Ice temperature at 1000 m depth at Talos Dome is ca. −25.5 ± 0.1 °C, while at the core bottom it is −8.9 ± 0.5 °C (Rix & Martin, personal communication). Increasing temperatures can accelerate both jarosite formation and dissolution^51^; however, over the full range of temperatures observed within the ice sheet, jarosite is considered stable^1,7,51^. Instead, the factor which enables the formation of jarosite below the depth of 1000 m, is likely the presence of liquid acidic brines and the concentration of impurities in the same isolated environment, where the pressure melting point is locally lowered^37–44^. This process is linked to ice metamorphism that is known to drive the migration and the concentration of impurities^39,40,42^. Once mineral particles and highly concentrated acidic fluids interact, chemical weathering of minerals is enhanced, allowing for the partial dissolution of original dust particles and the precipitation of new mineral phases^37,38,41,44,45^, such as jarosite. Reactions involving dust in deep ice should be investigated further in relation to the preservation of climatic signals in deep ice cores, in particular considering the “quest for the oldest ice”. Ice core records concerning the analysis of Fe concentration and speciation^52^ could be significantly affected by the processes described here.

Other implications of this study concern meteorites found at Antarctic blue ice fields. On the surface of such meteorites, a weathering rind 10–100 µm thick rich in jarosite and amorphous silica is commonly found, pointing to acidic alteration^18^. The rinds have been explained assuming that weathering occurred once meteorites were exposed at the ice surface, because of the interaction with acidic atmospheric species and tiny amounts of liquid water during summer^18^. Meteorites from blue ice can have remained into the ice for periods up to tens of thousands of years, reaching deep portions of the Antarctic ice sheet before surfacing^19,53,54^. The interface between deep ice and meteorites could promote the occurrence of acidic aqueous brines because of the crystallographic misfits between ice and mineral structures. Given the compositional similarity between the rinds and weathered dust in deep TALDICE, we suggest that englacial weathering should also be considered in relation to the chemical weathering of Antarctic meteorites, not only the surficial one. A support to this hypothesis comes from the Dome Fuji ice core, where jarosite has been found in correspondence of two extraterrestrial dust horizons, one interpreted as the result of a meteorite impact over East Antarctica, the other of the atmospheric entry of a fragment of an asteroid or a comet^55^. In TALDICE jarosite only forms below 1000 m deep; its presence in Antarctic meteorite rinds could be thus interpreted as a proxy of the englacial depth reached by meteorites themselves and of their residence time into the ice, as originally proposed by Terada et al.^19^.

### Martian implications

The occurrence of jarosite in TALDICE supports the ice-weathering model for the formation of Martian jarosite within large ice-dust deposits^14^. The environment inside the Talos Dome ice is isolated from the Earth atmosphere and its conditions, including pressure, temperature, pH and chemistry, provides a suitable analogue for similar Martian settings. Dust deposited at Talos Dome is also similar to Martian atmospheric dust, being both mostly basaltic^11,12^. Within thick ice deposits it is likely that the environment would be similar at Talos Dome and under Mars-like conditions since both settings would contain at cryogenic temperatures basaltic dust and volcanogenic and biogenic (for Antarctic only) sulfur-rich aerosols. What set them apart is the oxidative agent responsible for Fe oxidation. In deep TALDICE, Fe is likely oxidized through the interaction between air bubbles and clathrates and mineral dust. On Mars oxygen is likely derived from aerosols such as oxy-chlorine species formed through UV photo-oxidation^56^. Considering this context, it is reasonable that the formation of jarosite on Mars involves the interaction between brines and mineral dust in deep ice, as observed in TALDICE. This mechanism for Martian jarosite precipitation is paradigm changing and strongly challenges assumptions that the mineral formed in playa settings^3,5,11^.

Mars experienced dramatic climate swings and glaciations throughout its history^57,58^, and it has been argued that much of the physical layering observed on Mars surface could have been mediated by paleo ice-related processes^59^. The same could be for its mineralogical and geochemical features. In accordance with the ice-weathering model, Martian polar regions are rich in sulfate deposits which are created through the aeolian reworking of sublimation residues^60,61^. Despite these evidences and the Burns’ prophetic vision of Mars low-temperature, acid, water-limited alteration^1^, ice-mediated weathering has never been widely embraced or tested. Based on ongoing detections across the planet, jarosite is probably globally distributed across Mars in association with finely layered sediments^3–5^. Such sediments could result from the accumulation of weathered sublimation residue of past ice-rich deposits, since most of the settings where they occur are compatible with alteration pathways enabled by deep ice metamorphism and cryo-concentration of acidic fluids^14,15^. In contrast, recent studies have shown that the playa model does not adequately explain the chemical characteristics of jarosite rich deposits, their enormous size and the location of the putative playas^12,14,62^.

## Methods

### Dust concentration and grain size

Dust concentration and grain size in TALDICE have been determined through Coulter counter technique in the ISO6 clean room available at the EUROCOLD facility of the University Milano-Bicocca. Twenty-five cm-long ice sections were decontaminated with three successive baths in MilliQ water (^©^ Millipore) and mixed with a clean electrolyte solution (NaCl solution, passed through 0.22 µm pore size membranes) until a final Na^+^ concentration of 1% m/m was reached. The passage is required so as to make samples electrically conductive. Each sample, consisting in 10 mL of decontaminated meltwater, was measured three times with a Beckman Multisizer 4 equipped with a quartz tube with a 30 µm orifice. Such an instrumental apparatus allows for the quantification of the insoluble particles with a spherical equivalent diameter between 0.6 and 18 µm, divided into 400 channels. Volume of insoluble particles was then converted into mass using a constant density of 2.5 g cm^−3^, following the convention suggested by Hänel^63^ for mineral aerosols and classically adopted by the ice core community^64,65^. Blanks were constantly evaluated during the analysis, their signal, on average, corresponded to 2.5% of the mean sample one. Standard deviation of the replicates ranged between 5 and 10% with respect to the total dust mass of samples. Data concerning the highest part of TALDICE (0–900 m depth interval) have already been published^26,66^, data from the lowest part (from 900 m to 1616 m) are published here for the first time.

### Dust grain size index

To highlight the granulometric differences between dust samples from the upper and lower sections of TALDICE, a dedicated index was developed. For each dust sample analyzed with Coulter counter, sixteen granulometric variables were defined, consisting in dust concentrations and ratios referred to specific grain size intervals (Supplementary Information). Surficial samples are typically fitted by a log-normal distribution, with modal values ranging between 1.8 and 2.2 µm, and present a limited tail of coarser particles (5–10 µm) related to the influence of local Antarctic dust sources^26,66^. Deeper samples show a coarser modal value (between 2 and 4 µm) but are poorly described by log-normal distributions because of the presence of tails of coarse particles and the scarcity of finer ones (<2 µm). Examples are reported in the Supplementary Fig. 7. A Partial Least Square – Discriminant Analysis algorithm^67^ was applied to highlight such differences. Samples were firstly classified into upper (0–1450 m depth interval) and lower (1450–1620 depth interval), the algorithm was therefore instructed to reproduce the classification using the descriptive granulometric variables calculated from Coulter counter data, without considering the depth of samples. The dust grain size index shown in Fig. 1d corresponds to the linear combination of the granulometric variables defined by the first latent component calculated by the model to classify the samples. Further details are found in the Supplementary Information.

### X-ray absorption spectroscopy

Fe K-edge absorption energy was determined through XAS, performed at B18 beamline of the Diamond Light Source^68^. Analyses were carried out on samples prepared extracting mineral particles from decontaminated meltwater obtained from TALDICE ice sections in an ISO6 clean room. The extraction took place with clean polycarbonate membranes (rinsed with high purity nitric acid, pore-size 0.4 µm) where meltwater was passed through with micro-pipettes, so as to concentrate the particles in the smallest possible area and maximize the performances^69^. For each sample a variable volume of meltwater was filtered depending on dust concentration (evaluated with Coulter counter), so as to have on each filter not less than 2–3 µg of dust. Membranes were then placed in clean PTFE filter-holder and mounted on the beamline. Fifty-four samples were prepared, covering the entire length of the ice core; details, including spectral data, are reported in the Supplementary Information. To avoid contamination and interference issues, the following procedures were adopted: 1- the experimental chamber was provided with a clean glove-box filled with pure nitrogen in order to handle and mount the samples in clean conditions; 2- the inner walls of the chamber were coated with clean plastic sheets to reduce the inelastic scattering produced by the interaction of the beam with the metallic chamber; 3- measurements were carried out under high vacuum conditions to remove atmospheric interferences. XAS spectra were acquired at the Fe K-edge absorption line in fluorescence mode, considering the energy interval between 7000 and 7500 eV and energy steps of 0.3 eV. The incident beam was defocused as much as possible to maximize the irradiation area, its size was 0.4 × 0.4 mm. A Vortex 4-elements silicon detector was used (spectral resolution 140 eV full width height maximum at the 5.9 keV Mn K-line). The energetic calibration was monitored through the simultaneous acquisition of the absorption edge spectrum of a metallic Fe foil. For each sample–foil couple, the first derivative peak of the Fe K-edge of the foil was centered at 7112.0 eV, corrections of up to 0.2 eV were necessary. At least three spectra were acquired for each sample during different sessions and average spectra were calculated. Blank filters were measured but no Fe signal was detected. Standards (SRM 2709a, NIST) were prepared following the same procedures adopted for the samples to evaluate the precision of the method, which is 0.2 eV. Spectra were analyzed with the software Athena^70^. To make them comparable, fluorescence signals were processed to obtain absorption coefficients, which were normalized considering the post-edge baseline^71^. The pre-edge region was normalized subtracting the pre-edge baseline. The energy position of the Fe K-edge absorption was defined as the energy of the main absorption edge at the height of 0.8 (normalized value) with respect to the post-edge baseline. Errors shown in Fig. 1c correspond to the standard deviation calculated considering the replicates, further details are found in Cibin et al.^72^.

### Jarosite quantification

Jarosite Contribution was estimated comparing the Fe-K edge spectral signature of the samples with one of the mineralogical standards^73^. Specimens of the following minerals were retrieved from the collection of the University Milano-Bicocca: biotite, chlorite, glaucophane, goethite, hematite, hornblende, jarosite, limonite, magnetite, muscovite, fayalite, pargasite, pyrite, schoerlite, siderite. Fragments of the minerals were powdered using a mortar and mixed with cellulose in pellets which were measured at B18 in transmission mode. Fe K-edge absorption spectra were processed following the steps adopted for samples. To determine the amount of jarosite within the Fe minerals present in TALDICE dust, a linear combination fitting approach was followed^73^: spectra of the mineral standards were used to reproduce the spectra of the samples through the calculation of linear combinations with the combinatoric package of the Athena software^70^, based on ordinary least square regression (OLS). For each sample spectrum, all the possible combinations of 4 standards were calculated, forcing the presence of goethite, being the most common Fe-oxide within aerosols^31^, in particular in cold environments^32,74^. The contribution of jarosite with respect to total Fe minerals in TALDICE (Fig. 1b), corresponds to the coefficients (expressed as %) associated with jarosite within each linear combination. The % coefficient varies between 0 and 100 and is interpreted as the relative amount of information present in sample spectra that is explained by jarosite signature. The associated error is only statistical and corresponds to the OLS standard error of jarosite regression coefficient. Further details are found in the Supplementary Information.

### Microscopy

SEM-EDX observations were carried out on samples of dust extracted from TALDICE decontaminated meltwater, using a Zeiss Gemini 500 Field-Emission SEM equipped with EDX (Quantax EDX 4000, Bruker). Insoluble mineral dust was extracted from meltwater by filtration in an ISO6 clean room with polycarbonate membranes (diameter 25 mm, pore size 0.44 µm). After filtration some membranes were coated with gold for morphological observations (SEM) and others with graphite, for elemental mapping of mineral particles (SEM-EDX).

Two samples were prepared for STEM-EDX analyses following the same procedure adopted for SEM-EDX. Dust particles from two deep sections of TALDICE (below 1500 m) were deposited on polycarbonate membranes through filtration and successively picked, embedded in low viscosity epoxy, and thin sections were placed on amorphous carbon support films and were analyzed using a JEOL 2500SE 200 kV field-emission scanning and transmission electron microscope equipped with a JEOL silicon drift detector for EDX. Diffraction data from multiple orientations were obtained from selected areas of the grains to confirm their identity. Quantitative EDX data were obtained from the same grains as the diffraction data and were quantified using standard thin-film analysis techniques with k-factors from mineral standards (Table S1).

## Supplementary information

Supplementary Information

## Data Availability

The datasets generated and analyzed to the aims of the current study are available in the PANGAEA open repository: 10.1594/PANGAEA.924109, 10.1594/PANGAEA.924113, 10.1594/PANGAEA.924114.
