# Supplementary material for: Jarosite formation in deep Antarctic ice provides a window into acidic, water-limited weathering on Mars
Source: Nat Commun. 2021 Jan 19;12:436. doi: 10.1038/s41467-020-20705-z (PMC7815727; doi:10.1038/s41467-020-20705-z)
Supplement: Supplementary file 1 — Supplementary Information [file 41467_2020_20705_MOESM1_ESM.pdf]

Supplementary Materials to "Jarosite in Antarctic ice provides a window into acidic, water-limited weathering on Mars"

Giovanni Baccolo[1,2*], Barbara Delmonte[1], P. B. Niles[3], Giannantonio Cibin[4], Elena Di Stefano[1,2,5], Dariush Hampai[6], Lindsay Keller[3], Valter Maggi[1,2], Augusto Marcelli[6,7], Joseph Michalski[8], Christopher Snead[9] and Massimo Frezzotti[10].

[1]Department of Environmental and Earth Sciences, University of Milano-Bicocca, 20126, Milan, Italy

[2]INFN, section of Milano-Bicocca, 20126, Milan, Italy

[3]NASA Johnson Space Center, TX 77058, Houston, USA

[4]Diamond Light Source, Harwell Science and Innovation Campus, Didcot OX11 0DE, UK

[5]Department of Physical, Earth and Environmental Sciences, University of Siena, 53100, Siena, Italy

[6]Laboratori Nazionali di Frascati, Istituto Nazionale di Fisica Nucleare, 00044 Frascati, Italy

[7]Rome International Center for Materials Science - Superstripes, 00185 Rome, Italy

[8]Department of Earth Sciences, University of Hong Kong, Hong Kong

[9]Jacobs, NASA Johnson Space Center, TX 77058, Houston, USA

[10]Department of Science, University Roma Tre, Rome, Italy

* corresponding author: giovanni.baccolo@unimib.it

Supplementary note 1: Jarosite quantification

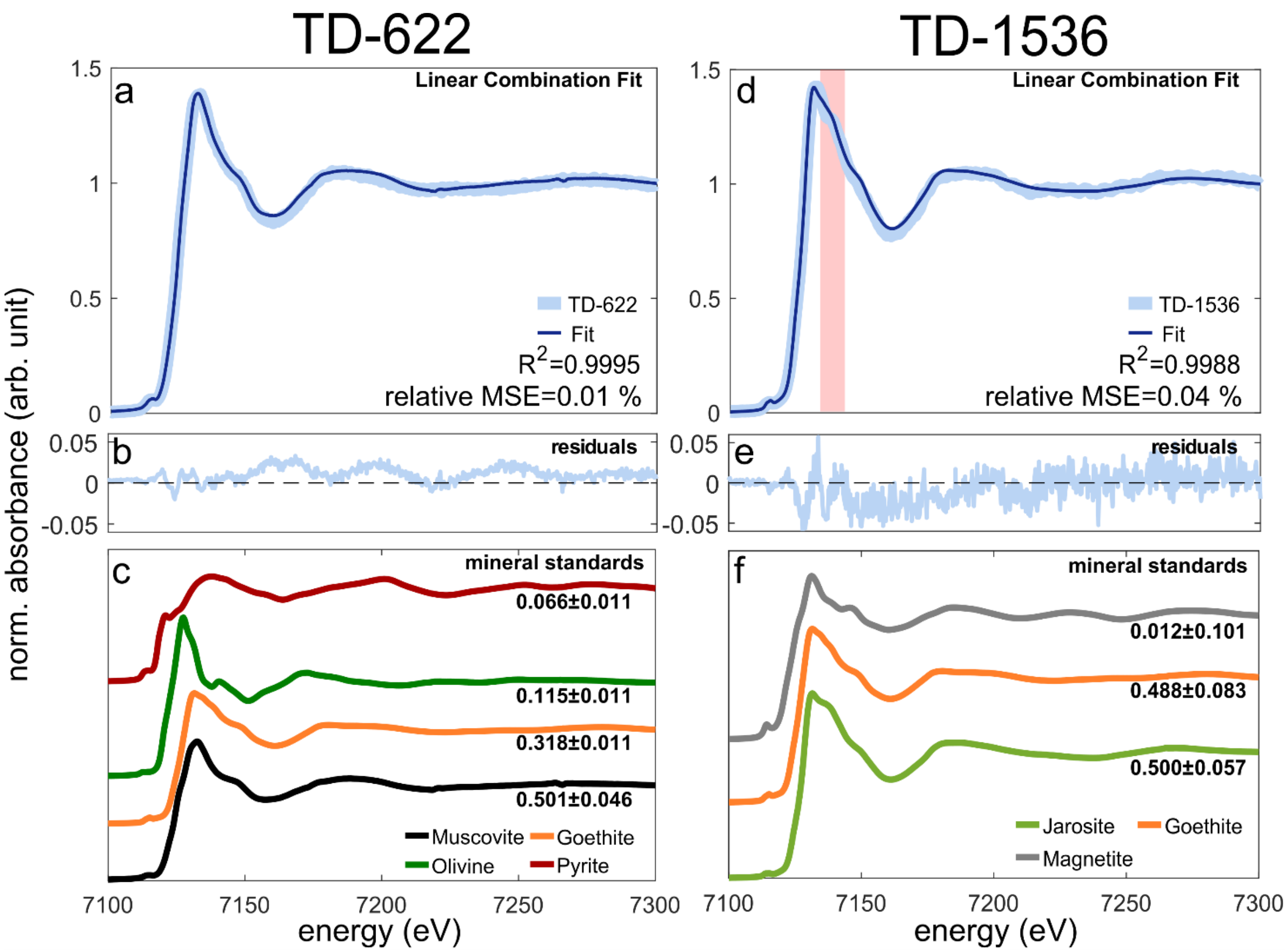

Supplementary Figure 1 Examples showing the method for the estimation of jarosite within TALDICE mineral dust samples through Fe K-edge absorption spectroscopy. The sample on the left (from a depth of 622 m) is from the upper part of the core, the sample on the right from the deep part (from a depth of 1536 m), where physical and chemical anomalies are found. The experimental spectrum of the two samples is shown in association to the one obtained through linear combination fitting (panel a and d), R-squared (R^2) and relative Mean Square Error (MSE) are also shown to evaluate the goodness of fit. The red square highlights the position of the spectral convexity (between 7135 and 7142 eV) typical of jarosite [1]. Residuals are shown in panels b and e. Panels c and f refer to the spectra of the mineral standards that are present within the linear combinations used to reproduce the spectra of the samples, the number reported with uncertainties correspond to the coefficients defining such linear combinations. Uncertainties are statistical only and correspond to the errors derived from the application of the Ordinary Least Square algorithm.

The following approach was adopted to quantify the jarosite contribute within the Fe fraction of mineral dust extracted from TALDICE ice through Fe K-edge X-ray absorption spectroscopy:

1. Dust samples and mineral standards were analyzed at B18 Diamond beamline. For each of them Fe K-edge X-ray spectra were acquired.
2. Spectra were processed following the same procedure with the Athena software so as to make them fully comparable [2]. For each sample, multiple spectra were acquired and averaged in order to increase the signal to noise ratio. Spectra were calibrated and then normalized with respect to pre- and post-edge baselines.

3. Linear combination fitting was applied to highlight the minerals whose Fe fraction displayed a spectral signature compatible with the sample ones, following the procedures adopted by Shoenfelt et al. [3] and Liu et al. [4]. For each sample all the possible linear combinations of 4 standards (15 mineral standards were considered) were calculated, including the combinations defined by 3,2 and 1 standards. To limit computing time an assumption was made, that is the presence of goethite among the 4 standards. This decision has been taken because goethite is an extremely common Fe oxide in mineral dust aerosol, in particular in cold environments [5, 6, 7]. Considering this point, 377 linear models were calculated for each sample.
4. The best linear combination was selected considering the associated R^2 value. It always exceeded 0.9, confirming that the minerals selected as standards were well representative of TALDICE dust.
5. The 4 coefficients defining the best combination for each sample were inspected and in particular the one for jarosite, which was interpreted as a proxy for jarosite relative abundance. The high R^2 values confirmed that the selected combinations were well representative of the samples, in other words the information present in sample spectra was well reproduced by the calculated linear combinations. Since the sum of the 4 coefficients defining each combination is 1, the single coefficients of each mineral standard can be interpreted as the relative amount of information explained by the considered standard. Hypothesizing that a sample is well reproduced (R^2 near 1) by a single mineral standard with a coefficient near 1 means that all the information present within the sample spectrum is easily reproduced using that single mineral. In contrast, all the mineral standards not selected to define the best combination, were avoided by the algorithm because they didn't bring an improvement to the model.
6. Considering the previous point, the index presented in Figure 1c as "Jarosite Contribution to Fe minerals" corresponds to the linear coefficient associated with jarosite within the best linear combination calculated for each sample. When the contribution is 0 (grey points in Figure 1c), it means that jarosite was not identified as one of the four best Fe-mineral standards to include within the model. When jarosite was considered, its linear coefficients were multiplied by 100 and reported in the graph as "Jarosite Contribution to Fe minerals (%)". The contribution thus corresponds to the relative amount of jarosite with respect to total Fe-bearing minerals present in TALDICE dust samples. It has to be intended as a relative quantity and not as an absolute estimate. The reason is that it only refers to the jarosite contribution with respect to total Fe present within the samples, not to total dust concentration.

Combining average data, it is possible to make a rough estimate of the amount of jarosite present in deep TALDICE between the depth of 1400 and 1620 m, where the XAS jarosite signal is maximum. Along this depth interval, the mean dust concentration is 140 ng g^{-1}_{ice}, corresponding to a total dust load in the 1400-1620 m ice column of 28 g m^{-2} (assumed dust density 2.5 g cm^{-3}). This datum is used to estimate the amount of Fe and of jarosite. To this am it is necessary to consider that previous data obtained through synchrotron radiation X-ray fluorescence showed that the Fe mass fraction of deep TALDICE dust is about 4.5 % [8]. This means that in the 1400-1620 m ice column, the total Fe associated with mineral dust corresponds to 1.3 g m^{-2}. In deep TALDICE the jarosite contribution to the Fe-related XAS signal is about 50 %. We thus obtain a rough estimate of jarosite content along the 1400-1620 m deep TALDICE ice column of 0.75 g m^{-2}. We note that this estimate is related to an environment, the Antarctic, where the amount of dust present in the atmosphere and in the ice is the lowest on Earth. Something very different characterizes Mars, where the concentration of dust in the atmosphere is extremely high and where the amount of dust in the putative ice-rich deposits would be orders of magnitude higher with respect to Antarctica [9].

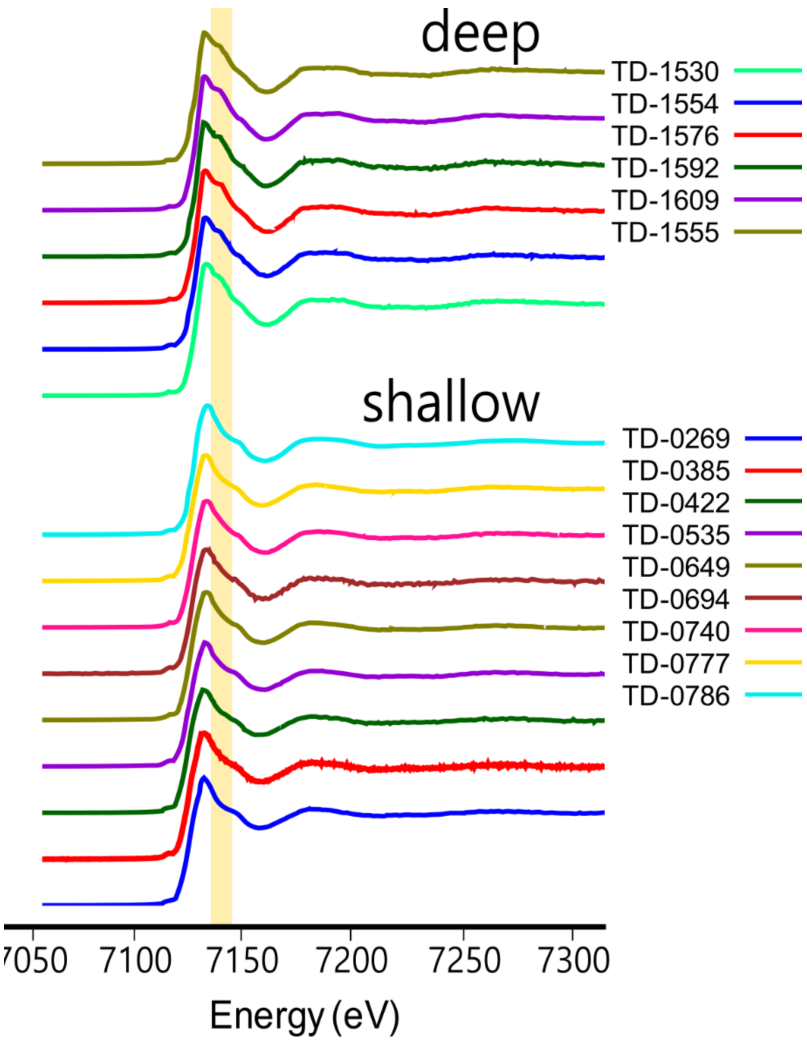

Supplementary Figure 2 Set of Fe K-edge XAS spectra concerning deep and shallow samples from the Talos Dome ice core (the energy interval between 7050 and 7300 eV is highlighted). The name of each sample refers to its depth along the core. It can be appreciated that the spectral rise characterizing jarosite between 7,135 and 7,142 eV (highlighted by the yellow band) is common in all the samples from the deepest part of the core, while it is never observed in the more surficial samples.

Supplementary note 2: Additional SEM-EDX observations

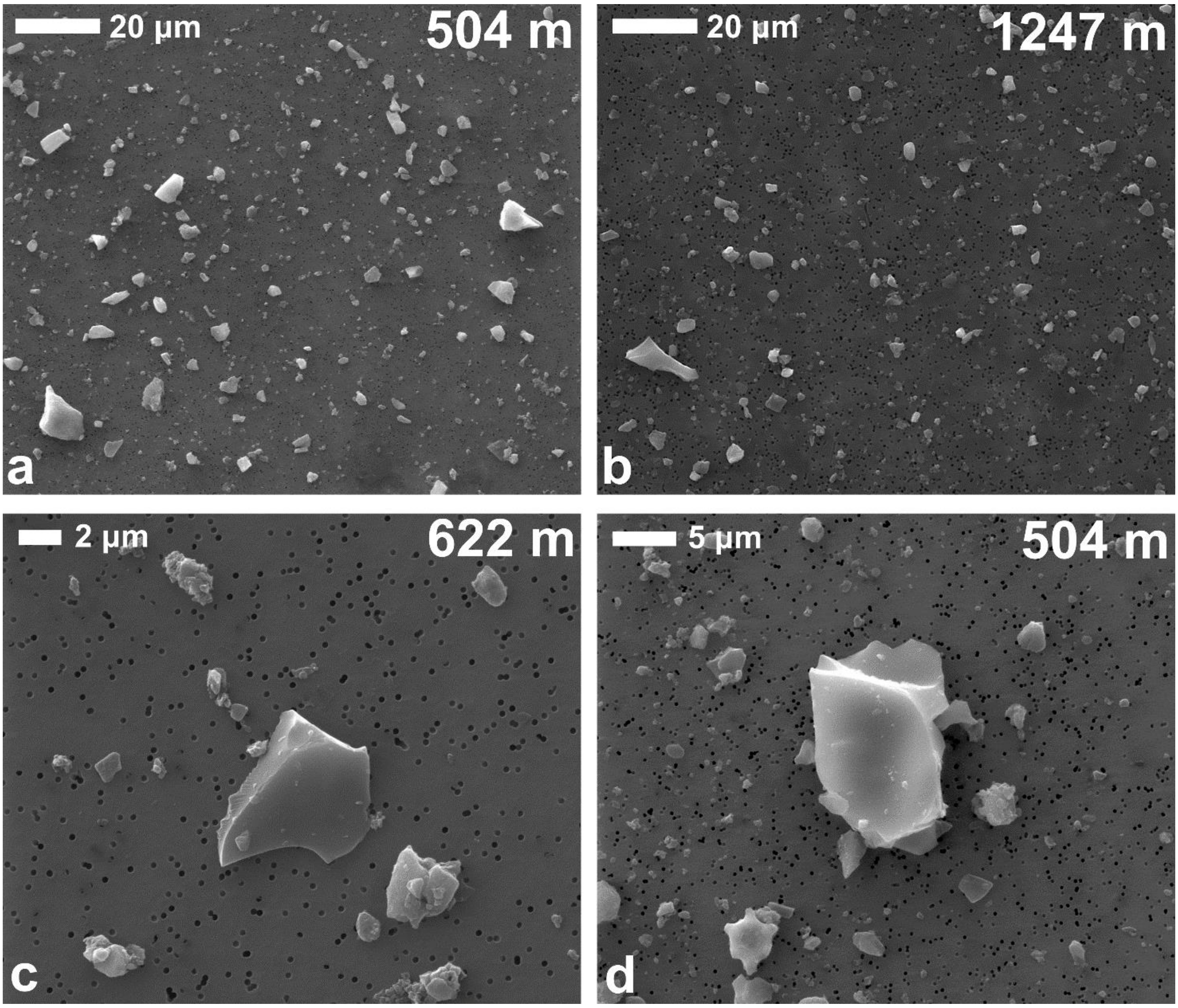

Supplementary Figure 3 SEM images of dust particles extracted from ice sections in the upper part of TALDICE, courtesy of Biancamaria Narcisi. The morphological features of these particles are different from the ones which characterize the particles from the deepest part of TALDICE, for a comparison see Figure 2 in the main text. These images were acquired in the context of a previous work on TALDICE atmospheric mineral dust [10].

The mineral particles that are present in the upper part of TALDICE, where the mineral dust record is not affected by significant post-depositional processes, show a morphology well distinct from the one characterizing the particles from the deepest part of the core. As appreciated in Supplementary Figure 3, mineral particles from the upper TALDICE sections mostly consist in volcanic shards and mono-mineral fragments with sharp edges and fresh textures. They are not aggregates of particles and lack weathering features as rounded edges, holes and depressions, which are common features in mineral particles entrapped in deep TALDICE (Figure 2 and Supplementary Figure 4). Details about the construction of elemental maps through SEM-EDX is shown in Supplementary Figure 5.

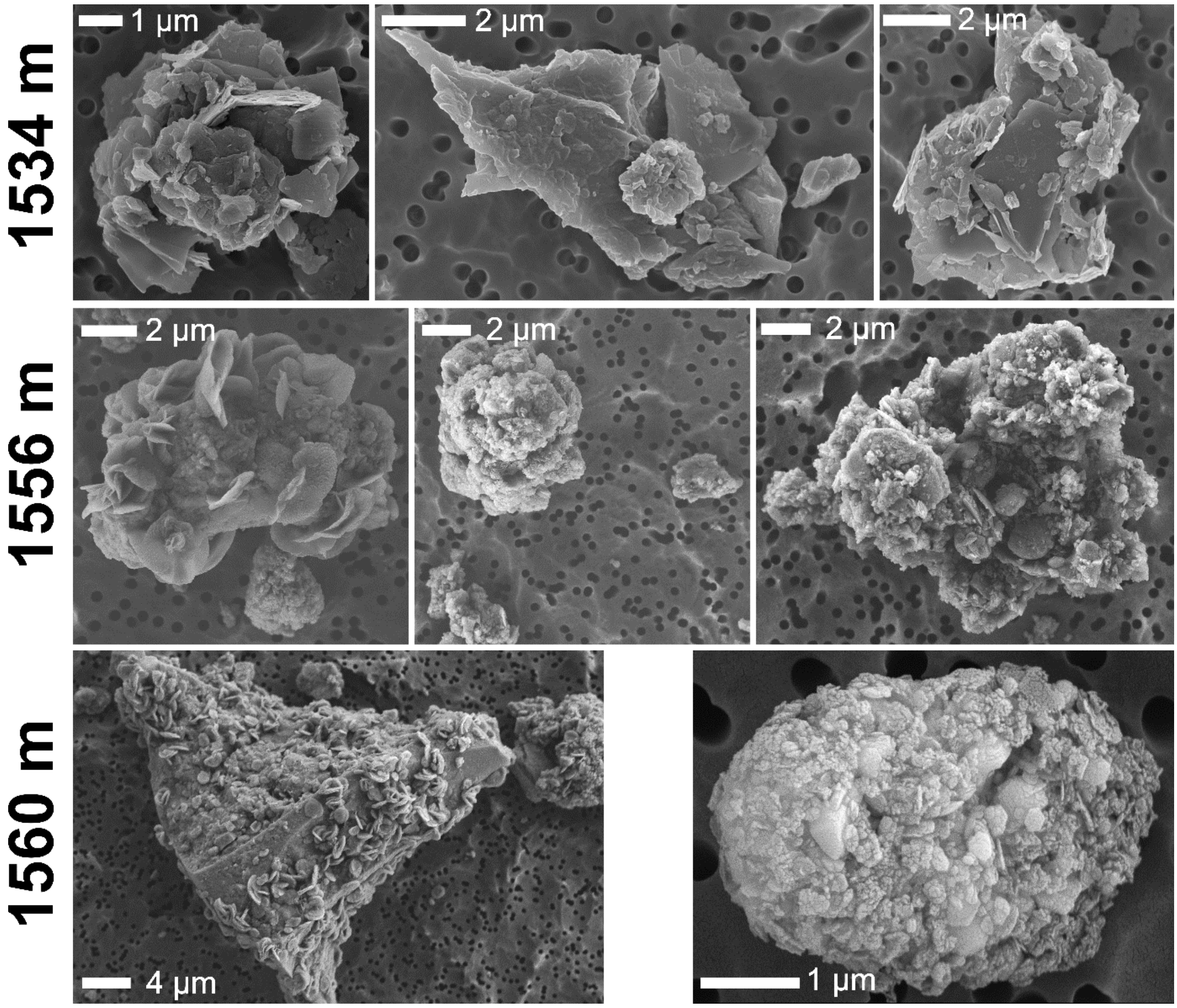

Supplementary Figure 4 Additional SEM images of dust particles extracted from deep TALDICE samples.

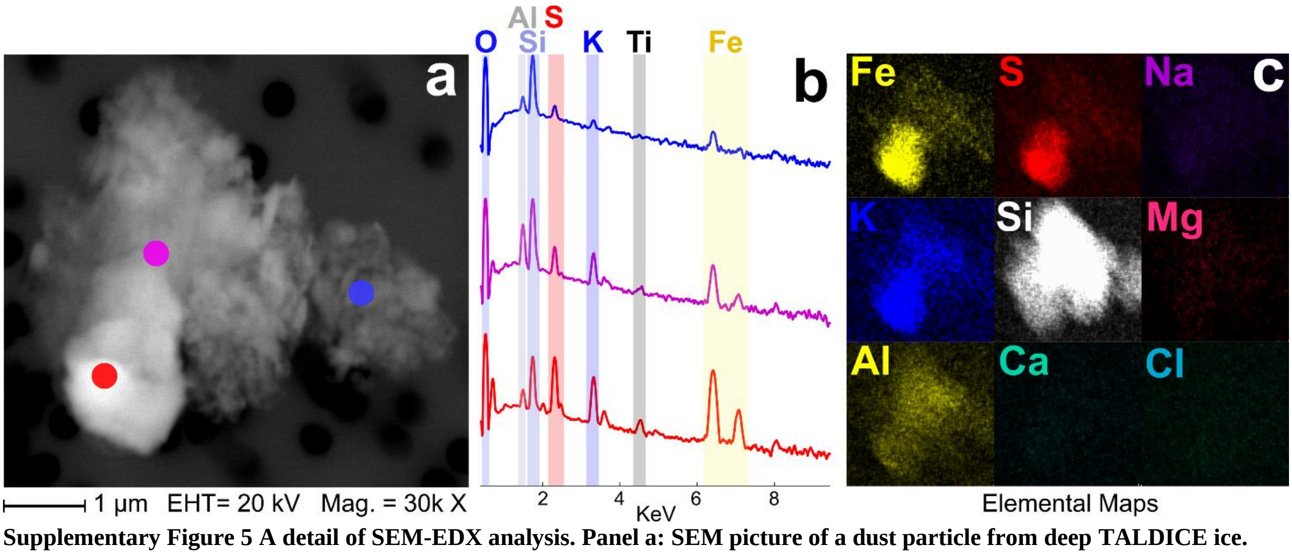

Supplementary Figure 5 A detail of SEM-EDX analysis. Panel a: SEM picture of a dust particle from deep TALDICE ice. Panel b: 3 EDX spectra related to the areas of the particle highlighted by dots in panel s (paired colors). Major peaks are associated to the elements responsible for their emission with colored bands. Panel c: elemental maps of the particle. It can be seen that the brighter grain on the left (red dot) is mostly composed by S, Fe and K, compatible with jarosite; the core of the particle is richer in Si and Al, a composition pointing to amorphous silica with substituted Al.

Supplementary note 3: TEM-EDX quantitative results

The two mineral grains investigated through TEM have also been analyzed with quantitative EDX spectroscopy to unveil their elemental composition. Results are shown in Supplementary Table 1: it is noted that their composition is perfectly compatible with the jarosite one. Major diffraction spacings from the [-110] zone axis of jarosite also confirm the identification of jarosite. They are: (003) 0.575 nm, (110) 0.365 nm and (113) 0.308 nm, all compatible with the well known diffraction features of jarosite [11].

Element Wt.%	Grain 1 Wt.%	Grain 2 Wt.%	Stoichmetric jarosite
H	n.a.	n.a.	1.21
O	41.3	41.2	44.72
Al	0.5	0.7	-
Si	0.7	1.1	-
P	1.4	1.4	-
S	14.4	14.1	12.81
K	7.0	6.7	7.81
Ca	0.0	0.1	-
Ti	1.3	0.6	-
Fe	33.5	34.2	33.45
Total	100.0	100.0	100.00

n.a. – not analyzed

Supplementary Table 1 EDX elemental composition of the grains identified as jarosite through TEM. A comparison shows a good stoichiometric correspondence with a jarosite reference.

Supplementary note 4: Correlation study

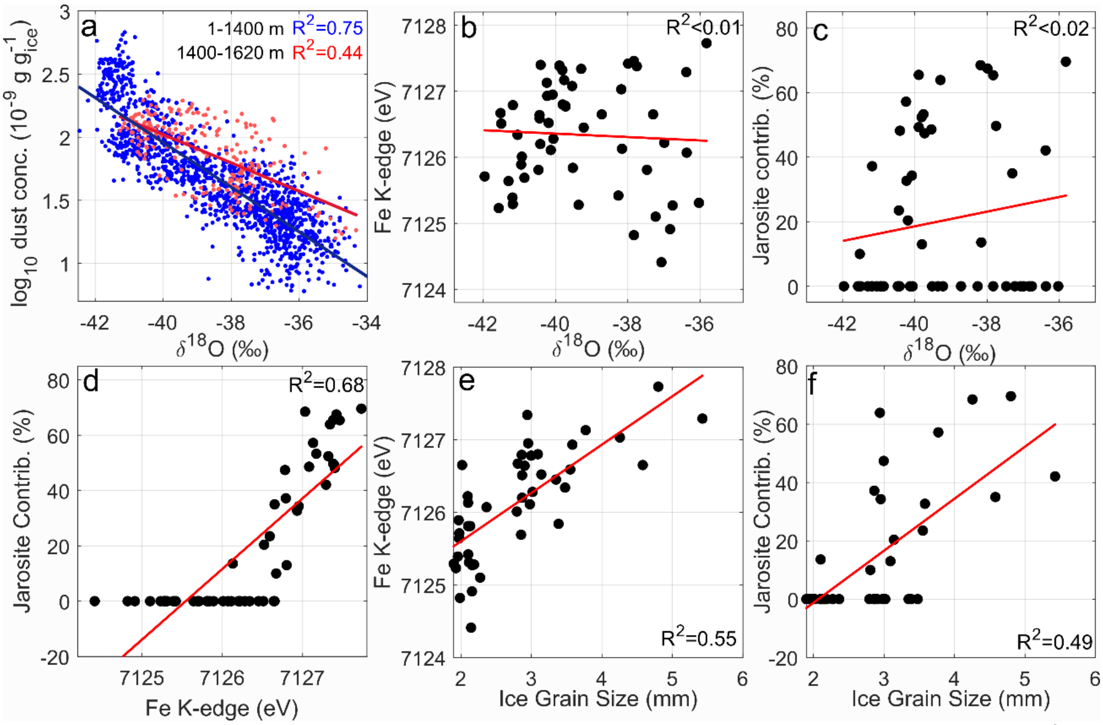

Supplementary Figure 6 Linear correlation between the TALDICE records considered in this study. a) water $\delta^{18}O$ (data from Stenni et al. [12]) vs. log_{10} of dust concentration within ice considering the shallow part of TALDICE (blue dots) and the deepest one (below 1400 m, red dots); part of the data from [13, 14]. b) water $\delta^{18}O$ vs. Fe K-edge absorption energy of mineral dust. c) water $\delta^{18}O$ vs. jarosite contribution in dust samples. d) Fe K-edge absorption energy vs. jarosite contribution in dust samples. e) Fe-K edge absorption energy vs. the size of ice grain size along the TALDICE core (data from Montagnat et al. [15]). f) Jarosite contribution in TALDICE dust samples vs. the size of ice grain size along the TALDICE core.

Supplementary note 5: Grain-size distributions

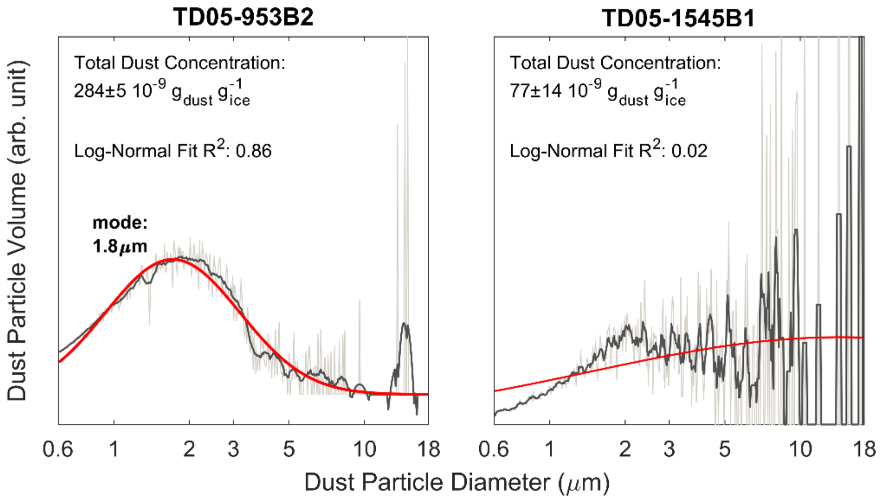

Supplementary Figure 7 Examples of granulometric distributions from TALDICE dust samples from Coulter counter analysis. On the left a sample from the shallow part of the core (953 m deep), where geochemical anomalies are not observed. The grain size is well reproduced using a log-normal distribution, a common feature of mineral dust extracted from East Antarctica ice [16] and indicative of prolonged atmospheric transport before deposition. On the right a sample from the deep part of the core (1545 m deep), where Fe oxidation and jarosite are found. The log-normal distribution is not suited for these samples, where an excess of dust coarser than 5 µm and a lack of the one smaller than 2 µm are observed with respect to typical Antarctic grain size distributions.

Supplementary note 6: Development of the "dust grain size" index

To develop the "grain size" index which highlighted the granulometric differences between dust samples from the upper and lower part of TALDICE, a Partial Least Square algorithm (PLS-DA) was applied [17]. The starting point for the application of the algorithm was the preparation of 16 variables defined to describe the 1517 samples measured through Coulter counter, they are:

1. Concentration within the 0.6-1.0 µm size interval
2. Concentration within the 1.0-2.0 µm size interval
3. Concentration within the 2.0-3.0 µm size interval
4. Concentration within the 3.0-4.0 µm size interval
5. Concentration within the 4.0-5.0 µm size interval
6. Concentration within the 0.6-5.0 µm size interval
7. Concentration within the 5.0-10.0 µm size interval
8. Concentration within the 0.6-10.0 µm size interval
9. Coarse local particle percentage (CLPP): $\frac{dust\ conc._{5-10\ \mu m}}{dust\ conc._{0.6-10\ \mu m}}$
10. Fine particle percentage (FPP): $\frac{dust\ conc._{0.6-2\ \mu m}}{dust\ conc._{0.6-5\ \mu m}}$
11. Coarse particle percentage (CPP): $\frac{dust\ conc._{3-5\ \mu m}}{dust\ conc._{0.6-5\ \mu m}}$
12. Concentration within the 10.0-18.0 µm size interval
13. $\frac{dust\ conc._{0.6-3\ \mu m}}{dust\ conc._{5-10\ \mu m}}$
14. $\frac{dust\ conc._{0.6-2\ \mu m}}{dust\ conc._{5-10\ \mu m}}$
15. $\frac{dust\ conc._{0.6-1\ \mu m}}{dust\ conc._{5-10\ \mu m}}$
16. $\frac{dust\ conc._{0.6-3\ \mu m}}{dust\ conc._{10-18\ \mu m}}$

After having defined these variables, samples were divided into shallow and deep in accordance to their depth: class "shallow" was applied to samples above 1450 m, "deep" to samples below 1450 m, where the geochemical and physical anomalies are evident. A first evaluation about the role of each one of the 16 variables with respect to the classification of the samples was carried out through multiple linear regression. Seventeen linear models were calculated: 1 considering all the variables and 16 excluding one variable at a time. For each of the models the associated R-squared

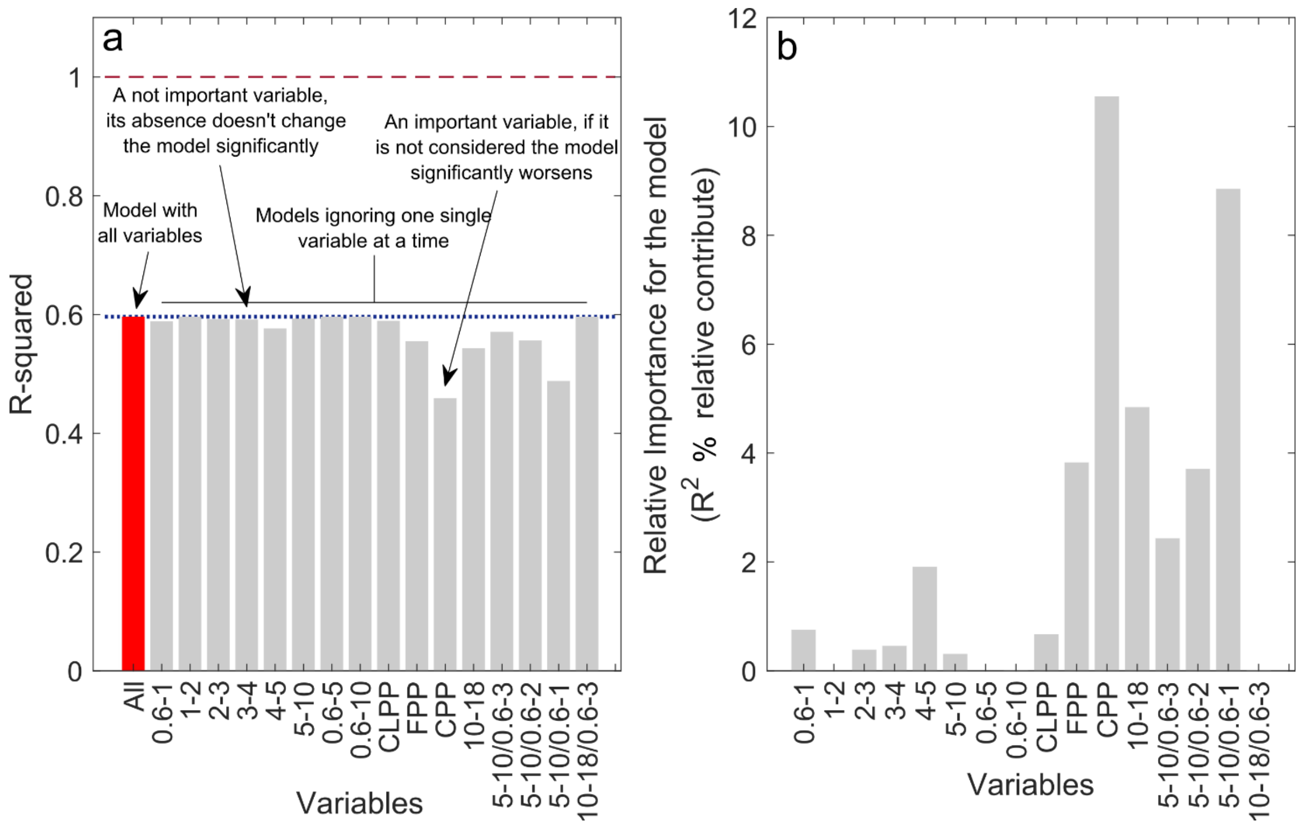

Supplementary Figure 8 Multiple linear models calculated to classify shallow and deep TALDICE dust samples on the basis of their granulometric features. Panel a: R-squared associated to the 17 calculated models. Panel b: the relative contribution of the single variables with respect to the performances of the complete model. It is expressed in terms of relative difference of R-squared related to the complete model and the one associated with the models calculated excluding the single variables.

was determined, results are shown in Supplementary Figure 8 as absolute R-squared values (panel a) and relative (%) contribution of each variable in determining the R-squared coefficient of the complete model. The complete model has an R-squared coefficient of 0.59 and the most important variables are CPP and the ratio between the dust concentration of the 5-10 and 0.6-1 µm size intervals. Other variables, such as FPP, the concentration of the particles between 10 and 18 µm, the 5-10/0.6-3, 5-10/0.6-2 µm interval ratios have a secondary role, while the other are negligible (Supplementary Figure 8b).

After this evaluation, the PLS-DA algorithm [17] was applied to reproduce the distinction between “shallow” and “deep” samples, still considering the 16 defined variables as descriptors. The R-squared of the obtained model was 0.63, meaning that the model could correctly reproduce 63% of the variance present within sample data. Another way to appreciate the performance of the model is the confusion matrix, where the actual classification is compared to the predicted one (Supplementary Table 2). In the table it is appreciated that 1400 of the 1401 “shallow” samples (99.9 %) and 78 of the 116 “deep” samples (65.5 %) were correctly classified.

The “dust grain size” presented in Figure 1d corresponds to the PLS component which showed the maximum relative ability to correctly classify the samples among the 16 calculated ones. In Supplementary Figure 9 the loadings of such component are shown in order to understand how the original variables contributed to the definition of the “dust grain size” index. It can be seen that the most important ones are the ratios between coarse and fine particle fractions and CPP (positive correlation with the index) and the FPP index (negative correlation).

		Predicted Classes		
		**Upper**	**Lower**	**Total**
True Classes	**Upper**	1400	1	1401
	**Lower**	38	78	116
	**Total**	1438	79	

Supplementary Table 2 Classification performances of the PLS-DA algorithm represented through the confusion matrix, where actual and predicted classes are compared.

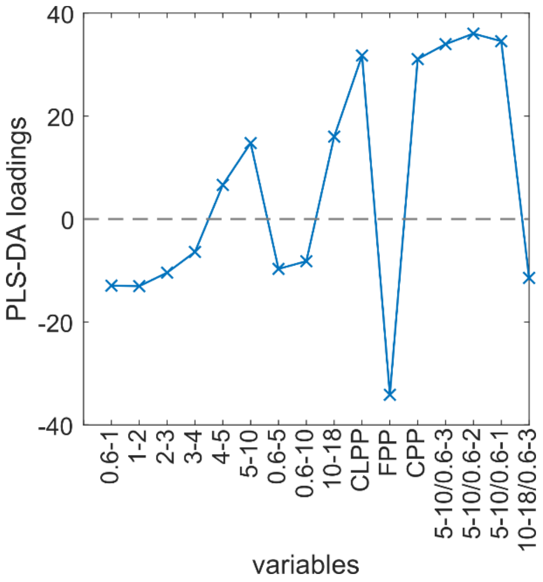

Supplementary Figure 9 The loadings of the PLS-DA component which correspond to the "dust grain size" index presented in Figure 1d.

Supplementary References

[1] Johnstone, S. G. et al. Arsenic mobilization and iron transformations during sulfidization of As (V)-bearing jarosite. *Chem. Geol.* **334**, 9-24 (2012).

[2] Ravel, B. & Newville, M. ATHENA, ARTEMIS, HEPHAESTUS: data analysis for X-ray absorption spectroscopy using IFEFFIT. *J. Synchrotron Radiat.* **12**, 537-541 (2005).

[3] Shoenfelt, E. M., Winckler, G., Lamy, F., Anderson, R. F., Bostick, B. C. Highly bioavailable dust-borne iron delivered to the Southern Ocean during glacial periods. *PNAS* **115**, 11180-11185 (2018).

[4] Liu, S. et al. Iron Speciation in Insoluble Dust from High-Latitude Snow: An X-ray Absorption Spectroscopy Study. *Cond. Matter.* **3**, 47 (2018).

[5] Formenti, P. et al. Dominance of goethite over hematite in iron oxides of mineral dust from Western Africa: Quantitative partitioning by X-ray absorption spectroscopy. *J. Geophys. Res. Atmos.* **119**, 12740-12754 (2014).

[6] Zhang, X. L., Wu, G. J., Zhang, C. L., Xu, T. L., Zhou, Q. Q. What is the real role of iron oxides in the optical properties of dust aerosols?. Atm*os. Chem. Phys.* **15**, 12159-12177 (2015).

[7] Cong, Z. et al. Iron oxides in the cryoconite of glaciers on the Tibetan Plateau: abundance, speciation and implications. *Cryosphere* **12**, 3177-3186 (2018).

[8] Baccolo, G. et al. The Contribution of Synchrotron Light for the Characterization of Atmospheric Mineral Dust in Deep Ice Cores: Preliminary Results from the Talos Dome Ice Core (East Antarctica). *Condensed Matt.* **3**, 25 (2018).

[9] Tanaka, K. L. Dust and Ice Deposition in the Martian Geologic Record. *Icarus* **144**, 254-266 (2000).

[10] Delmonte, B. et al. Modern and Holocene aeolian dust variability from Talos Dome (Northern Victoria Land) to the interior of the Antarctic ice sheet. *Quaternary Sci. Rev.* **64**, 76-89 (2013).

[11] Baron, D. & Palmer, C. D. Solubility of jarosite at 4-35 °C. *Geochim. Cosmochim. Ac.* **60**, 185-195 (1996).

[12] Stenni, B. et al. Expression of the bipolar see-saw in Antarctic climate record during the last deglaciation. *Nat. Geosci.* **4**, 46-49 (2011).

[13] Albani, S. et al. Interpreting last glacial to Holocene dust changes at Talos Dome (East Antarctica): implications for atmospheric variations from regional to hemispheric scales. *Clim Past* **8**, 741-750 (2012).

[14] Baccolo, G. et al. Regionalization of the atmospheric dust cycle on the periphery of the East Antarctic ice sheet since the last glacial maximum. *Geochem. Geophy. Geosy.* **19**, 3540-3554 (2018).

[15] Montagnat, M. et al. Measurements and numerical simulation of fabric evolution along the Talos Dome ice core, Antarctica. *Earth Planet. Sci. Lett.* **357-358**,168-178 (2012).

[16] Delmonte, B., Petit, J. R., Maggi, V. Glacial to Holocene implications of the new 27000-year dust record from the EPICA Dome C (East Atarctica) ice core. *Clim, Dynam*, **18**, 647-660 (2002).

[17] Ballabio, D. & Consonni, V. Classification tools in chemistry. Part 1: Linear models. PLS-DA. *Anal. Methods* **5**, 3790-3798 (2013).
